# Elderly Patient With Hematological and Neurological Manifestations of Undetermined Origin: A Diagnostic Dilemma of Pernicious Anemia

**DOI:** 10.7759/cureus.43045

**Published:** 2023-08-06

**Authors:** Anas Ibraheem, Abdulqadir J Nashwan, Mohamed A Yassin

**Affiliations:** 1 Internal Medicine, Imamein Kadhimein Medical City, Baghdad, IRQ; 2 Internal Medicine, Clinical Hematology, Al Karama Teaching Hospital, Baghdad, IRQ; 3 Nursing, Hamad Medical Corporation, Doha, QAT; 4 Hematology, National Centre for Cancer Care and Research, Hamad Medical Corporation, Doha, QAT

**Keywords:** multidisciplinary care approach, diagnosis challenges, subacute combined degeneration, vitamin b12 deficiency, pernicious anemia

## Abstract

Pernicious anemia (PA) is a chronic inflammatory destructive disease of parietal cells of predominantly the gastric fundus. It leads to vitamin B_12_ (cobalamin) deficiency secondary to a deficiency of intrinsic factors. Despite the medical advances nowadays, diagnosing PA can be challenging. This report highlights a neglected case of PA with ongoing subacute combined degeneration of the cord (SCDS) in an elderly patient. An 86-year-old lady with multiple comorbidities was referred to the hematology outpatient clinic for refractory anemia for the last two months. At first, her general practitioner (GP) treated her as a case of anemia of chronic disease but without improvement. Her initial clinical assessment revealed hematological and neurological manifestations of undetermined origin, including global weakness, hypertonia, and hyperreflexia with sensory deficits, especially in the lower limbs. On investigation, the hemoglobin level was 9 g/dL with high indirect bilirubinemia and lactate dehydrogenase (LDH). Despite the normal mean corpuscular volume (MCV) and peripheral blood smear, positive anti-intrinsic factor and parietal cell antibodies tests were subsequently reported, suggesting the diagnosis of PA. As a result, she was commenced on lifelong parenteral cobalamin replacement therapy. On follow-up visits of three months, she illustrated a clinical recovery in fatigability and paranesthesia. As expected, the laboratory parameters revealed a rise in hemoglobin level (11 g/dL) and serum vitamin B_12_ (418 pg/mL). However, she remained bedridden with spastic limbs. Clinicians should have a high index of suspicion since PA is a rare disease with variable clinical presentations. The optimal management approach is by a multidisciplinary care team of internists, neurologists, gastroenterologists, and hematologists.

## Introduction

Pernicious anemia (PA) is a chronic inflammatory destructive disease of parietal cells of predominantly the gastric fundus. It leads to vitamin B_12_ (cobalamin) deficiency secondary to a deficiency of intrinsic factors. Normally, this factor binds to vitamin B_12_ and facilitates its transport and absorption in the ileum [1-2]. PA is a rare disease with only 0.1% prevalence in the general population. The higher prevalence with age reaches up to 1.9% in patients older than 60. Up to half of the cases of vitamin B_12_ deficiency in adults are caused by PA [3]. Both genders are equally affected, with some variation among different geographical regions [1]. To date, the diagnosis of PA is often unrecognized and probably missed because of the highly variable clinical presentation of the disease and the unavailability of reliable diagnostic tests [1]. PA is a treatable disease; however, the consequences can be devastating if undiagnosed [1]. Therefore, clinicians must have a high index of suspicion. A triad of undetermined hematological and neurological manifestations, low serum B_12_, and response to parenteral vitamin B_12_ replacement therapy is essential in supporting the diagnosis [4]. If untreated, PA can result in subacute combined degeneration of the cord (SCDS), a dreadful neurological consequence of cobalamin deficiency. SCDS is characterized by degeneration of the dorsal and lateral columns of the spinal cord due to demyelination. A typical presentation of the disease is sensory deficits, paresthesia, weakness, ataxia, and gait disturbance. Spasticity and paraplegia can be the fate of severely untreated patients [5-6]. This report highlights a neglected case of PA with ongoing subacute combined degeneration of the cord in an elderly patient with multiple comorbidities.

## Case presentation

An 86-year-old lady with a past medical history of hypertension, congestive heart failure (CHF) on a background of ischemic heart disease, primary pulmonary hypertension (PPH), and a stage 3 non-dialysis-dependent chronic kidney disease (CKD) was referred to the hematology outpatient clinic due to refractory anemia for the last two months. Her general practitioner (GP) treated her as a case of anemia of chronic disease. Although epoetin alfa injections (2000 units/mL) were subcutaneously administered three times weekly, anemia did not improve. That said, she did not receive any blood transfusion during that period.

At the presentation, being bedridden with a side complaint of paresthesia in her fingers and toes was vaguely explained since she had no previous cerebrovascular accidents, head or spine trauma, tumor, or surgery. Her condition appeared more complicated than the reason behind the referral. Therefore, a more detailed history was taken.

She was a non-smoker and non-alcoholic with a modest eating habit of only a little meat, fish, eggs, and vegetables almost daily. She denied any gastrointestinal symptoms such as loss of appetite, diarrhea, hematochezia, or steatorrhea. However, she lost 10 kg of weight over the last five months. Otherwise, there was no history of fever or night sweats. Her home medications included sacubitril/valsartan 24 mg/26 mg, carvedilol 12.5 mg, digoxin 125 mcg, furosemide 40 mg, atorvastatin 20 mg, folic acid 5 mg, One Alpha 0.25 mcg, apixaban 2.5 mg, and tadalafil 10 mg.

Six months ago, she was admitted to the hospital based on a provisional diagnosis of PPH. She was treated accordingly and discharged on tadalafil and short-term oxygen therapy. Thus, she was bedbound by avoiding any physical activity, including walking. As a result, she became bedridden despite attempts of rehabilitation and exercise to restore her muscle bulk.

The family lost any hope that she could walk again. Unfortunately, any afterward complaint was blamed on senility and being bedridden; therefore, her anemia was underestimated.

Her initial clinical assessment revealed a conscious-oriented lady with pale conjunctiva, a smooth-large tongue, generalized weakness, and loss of all muscle bulk. On the other hand, she did not have jaundice or organomegaly or lymphadenopathy. No signs of bleeding were noted in any part of her physical examination. Over and above, her vital signs were unremarkable.

A meticulous neurological examination revealed intact cognitive function (Mini-Mental State Examination score of 28) with spastic upper and lower limbs on both sides. In addition, brisk deep biceps, triceps, patellar, and Achilles tendons reflexes were elicited, besides the corresponding clonus. Hoffmann and Babinski's signs were bilaterally positive. The most prominent weakness was in the lower limbs, with a muscle power of two; as a result, her gait could not be assessed. Interestingly, mild ataxia was noticed throughout her lower limbs examination.

Light touch and vibration were impaired up to the level of the knees with absent proprioception. Otherwise, the rest of the assessment was unremarkable, including cranial nerves and cerebellar function.

**Figure 1:**
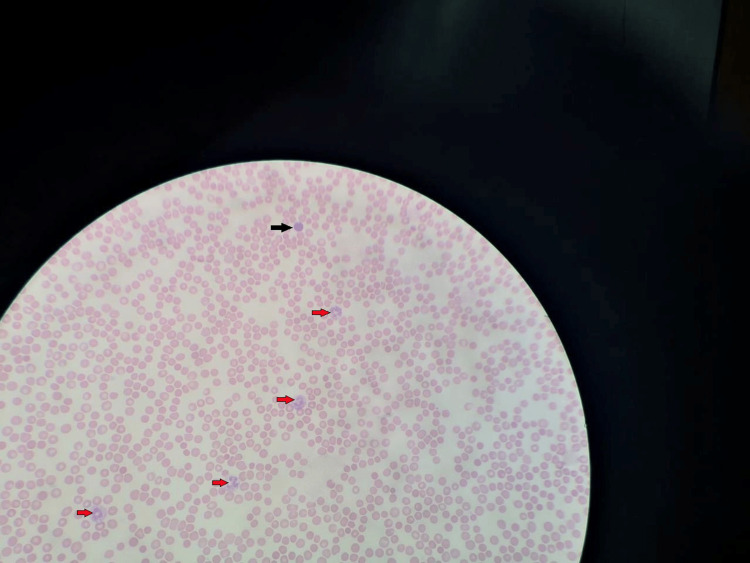
Patient’s peripheral blood smear showing a full field of normocytic-normochromic erythrocytes. Normal neutrophil cells (red arrow) and a small lymphocyte (black arrow) are also seen.

On investigation, her complete blood count showed a hemoglobin of 9 g/dL with unremarkable mean corpuscular volume (MCV) of 94 fL and almost other blood indices, including white blood count and platelet count. Peripheral blood smear showed normocytic-normochromic erythrocytes with normal neutrophil morphology (Figure 1).

Biochemical parameters were normal except for high indirect bilirubinemia and lactate dehydrogenase (LDH). A fecal occult blood test was negative. The plasma level of cobalamin was low (137 pg/mL), while the folate was normal. Interestingly, anti-intrinsic factors (anti-IFABs) and anti-parietal cell (APCA) antibodies were strongly positive (Table 1). Native and contrast-enhanced cranial and spinal cord MRIs were unremarkable except for senile changes.

**Table 1:** Summary of the patient’s laboratory data on admission. MCV, mean corpuscular volume; MCH, mean corpuscular hemoglobin; MCHC, mean corpuscular hemoglobin concentration; RDW, red cell distribution width; TIBC, total iron binding capacity; PT, prothrombin time; INR, international normalized ratio; PTT, partial thromboplastin time; ALP, alkaline phosphatase; ALT, alanine aminotransferase; AST, aspartate aminotransferase; T3, triiodothyronine; T4, thyroxine; TSH, thyroid stimulating hormone; BUN, blood urea nitrogen; Na, sodium; K, potassium; Cl, chloride; CRP, C-reactive protein; LDH, lactate dehydrogenase; anti-IFABs, anti-intrinsic factor antibodies; APCA, anti-parietal cell antibodies

Test	Result	Reference range	Test	Result	Reference range
White blood count	4.5×10^9^/L	4.5-11×10^9^/L	Fibrinogen	358 mg/dL	237-481 mg/dL
Neutrophile	69%	40-60%	D-dimer	3.4 μg/mL	≤0.49 μg/mL
Lymphocyte	24.7%	20-40%	Total protein	5.2 g/dL	6-8 g/dL
Monocyte	5.2%	2-8%	ALP	127 U/L	35-104 U/L
Eosinophil	1.1%	1-4%	ALT	42 U/L	10-35 U/L
Basophil	0%	0.5-1%	AST	55 U/L	10-35 U/L
Platelet count	152×10^9^/L	145-400×10^9^/L	Haptoglobin	163 mg/dL	30-200 mg/dL
Red blood cell count	3.18×10^12^/L	3.8-5.8×10^12^/L	T3	1.9 nmol/L	1.2-2.8 nmol/L
Hemoglobin level	9 g/dL	11.5-16 g/dL	T4	98 nmol/L	77-155 nmol/L
MCV	94 fL	80-100 fL	TSH	3.8 mIU/L	0.3-4 mIU/L
MCH	28.3 pg	27-32 pg	BUN	109 mg/dL	5-20 mg/dL
MCHC	32 g/dL	32-36 g/dL	Creatinine	1.3 mg/dL	0.5-1.1 mg/dL
RDW	15%	12-15%	Na	143 mmol/L	135-145 mmol/L
Reticulocyte	0.2%	0.6-2.4%	K	4.2 mmol/L	3.5-5 mmol/L
Serum vitamin B_12_ level	137 pg/mL	232-1245 pg/mL	Cl	102 mmol/L	96-106 mmol/L
Serum folate level	14.6 ng/mL	≥4.6 ng/mL	Serum erythropoietin	5.5 mU/mL	2.6-18.5 mU/mL
Serum iron level	110 μg/dL	37-145 μg/dL	CRP	1.2 mg/dL	0.3-1.0 mg/dL
Serum ferritin level	278 ng/mL	12-263 ng/mL	Total bilirubin	1.8 mg/dL	0-1 mg/dL
TIBC	362 μg/dL	250-430 μg/dL	Direct bilirubin	0.2 mg/dL	0-0.3 mg/dL
Transferrin saturation	30%	15-50%	Indirect bilirubin	1.6 mg/dL	0.2-0.8 mg/dL
PT	13.5 seconds	11.8-14.4 seconds	LDH	674 U/L	105-333 U/L
INR	1.15	0.87-1.13	Anti-IFABs	4.2 AU/mL	1.21-1.52 AU/mL
PTT	31.5 seconds	24.4-36.6 seconds	APCA	38 Units	0-20 Units

Her family refused invasive procedures such as gastroscopy to exclude possible differentials or prevalent lesions (carcinoid tumors and gastric cancer).

Based on the clinical course and the laboratory findings, she was treated as a case of pernicious anemia with ongoing subacute combined degeneration of the spinal cord. Consequently, intramuscular vitamin B_12_ injections of 1000 mcg were prescribed once daily for one week, then weekly for one month, and then monthly for lifelong. In addition, a neurologist referral was executed to assess and monitor the neurological deficits of the subacute combined degeneration.

On subsequent follow-up visits of three months, she illustrated a clinical recovery in fatigability and paranesthesia. As expected, the laboratory parameters revealed a rise in hemoglobin level (11 g/dL) and serum vitamin B_12_ (418 pg/mL). However, she remained bedridden with spastic limbs.

## Discussion

Despite the medical advances nowadays, diagnosing pernicious anemia can be challenging due to the complex nature of the disease, the broad spectrum of clinical presentation, and the unavailability of a gold standard test [1]. In this report, the GP treated the patient as a case of anemia of chronic disease based on her initial laboratory findings and the medical history of CKD. However, her anemia remained at a plateau level (∼9 g/dL) and was never corrected. The poor diet as a cause of the patient's anemia was unlikely, depending on her eating habits of foods rich with vitamin B_12_, including fish, meat, eggs, and dairy products. Of note, the vitamin B_12_ recommended dietary allowance for her age and gender is as minimum as 2.4 mcg/day [7].

Her subtle anemia and other hematological parameters prior to referral can be attributed to the effect of her daily consumption of folic acid (5 mg/day) as a part of her CKD treatment regimen. Various studies suggested that folic acid supplementation may mask an occult vitamin B_12_ deficiency, even at doses <1 mg/day. The outcome can change the presentation of the disease by correcting the hematological indices without neurological improvement. Respectively, it will delay the diagnosis of B12 deficiency leading to further exacerbation and/or the initiation of neurological diseases [8-11].

Although an MCV of more than 115 fL is considered more specific for B_12_ deficiency, normocytic anemia with an unremarkable peripheral blood smear should not rule out PA [1,5]. Therefore, PA must be considered a differential diagnosis in any patient with hematological and neurological manifestations of undetermined origin [3]. Different studies revealed that neurological deficit can occur in B_12_ deficiency with normal hematological indices, including MCV in 25% of cases [1,5,12]. On the other hand, plasma homocysteine and methylmalonic levels can be used to support the diagnosis if serum B_12_ levels are borderline [5]. In this case, it was avoided since the B_12_ level was low. Furthermore, the British Committee for Standards in Hematology recommended that all anemic patients with neuropathy or glossitis suspected of having PA should be tested for anti-IFABs regardless of cobalamin levels [12]. Even if the anti-IFABs test is highly specific for PA with a positive predictive value of 95%, it has a low sensitivity rate [13-14]. Thus, a negative test should not rule out PA if there is a high index of suspicion.

In our case, positive anti-IFABs and APCA tests were subsequently reported, suggesting the diagnosis of pernicious anemia. As a result, she was commenced on lifelong parenteral cobalamin replacement therapy [1,12]. Although vitamin B_12_ deficiency may mimic myelodysplastic syndrome and other hematological malignancies in clinical presentation, bone marrow aspiration/biopsy was not considered in our patient due to the absence of a trial of vitamin B_12_ therapy and failure of meeting Vienna's minimal diagnostic criteria for myelodysplastic syndrome [15-16]. In addition to a lifelong parenteral cobalamin replacement therapy, the management plan of such patients includes regular assessment and monitoring by a neurologist for the neurological deficits of SCDS and a referral for upper gastrointestinal endoscopy to exclude relevant malignancies (gastric carcinoid tumors and adenocarcinomas) [5,17-18]. Physical rehabilitation is encouraged to reduce spasticity and improve gait and balance control [5].

B_12_ supplementation can stop disease progression and improves hematological symptoms within days to weeks. However, the neurological recovery's magnitude is inversely proportional to the severity and duration of symptoms before treatment [1]. After starting treatment, better prognosis and neurological outcomes are associated with some characteristics, including being under 50 years, short-course disease, normal sensation, absent Romberg and Babinski sign, and less than seven medullary segments affected on MRI, and no medullary atrophy [19]. Reflecting on our case, the patient was over 50 with severe neurological deficits and a positive Babinski sign, which gave a poor neurological prognosis.

## Conclusions

This article highlights a case of pernicious anemia with an ongoing SCDS. PA is a treatable disease; on the contrary, if it is left undiagnosed, the consequences can range from irreversible neurological complications to death in serious cases. Therefore, clinicians should have a high index of suspicion since PA is a rare disease with variable clinical presentations. The optimal management approach is by a multidisciplinary care team of internists, neurologists, gastroenterologists, and hematologists. Those patients must remain on lifelong parenteral B_12_ injections and regular follow-ups.
